# Supplementary figures and images for: Evaluation of traditional and bootstrapped methods for assessing data-poor fisheries: a case study on tropical seabob shrimp (Xiphopenaeus kroyeri) with an improved length-based mortality estimation method
Source: PeerJ. 2024 Nov 14;12:e18397. doi: 10.7717/peerj.18397 (PMC11569787; doi:10.7717/peerj.18397)

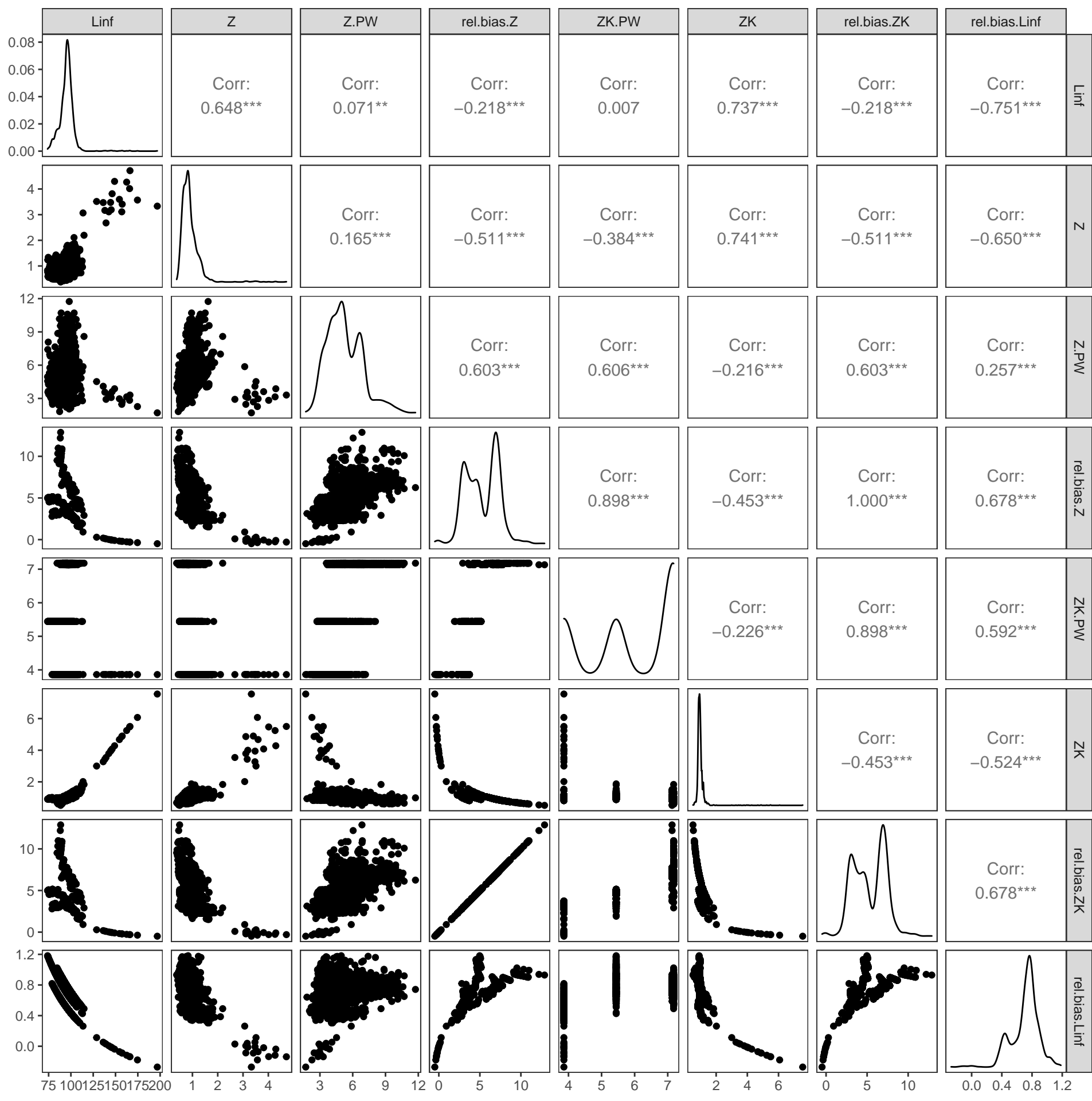

Supplement: Supplemental Information 2 — Scatterplots showing multivariate relationships between different parameter estimates [file peerj-12-18397-s002.pdf]
